# Extraction and Quantitation of Phytosterols from Edible Brown Seaweeds: Optimization, Validation, and Application

**DOI:** 10.3390/foods12020244

**Published:** 2023-01-05

**Authors:** Zhen Chen, Nianqiu Shen, Xunzhi Wu, Jiaping Jia, Yue Wu, Hitoshi Chiba, Shuping Hui

**Affiliations:** 1 Faculty of Health Sciences, Hokkaido University, Kita-12, Nishi-5, Kita-ku, Sapporo 060-0812, Japan; 2 Department of Nutrition, Sapporo University of Health Sciences, Nakanuma Nishi-4-2-1-15, Higashi-ku, Sapporo 007-0894, Japan

**Keywords:** hijiki (*Sargassum fusiforme*), wakame (*Undaria pinnatifida)*, kombu (*Saccharina japonica*), fucosterol, saringosterol, ostreasterol, ultrasound-assisted extraction, design of experiments

## Abstract

Brown seaweeds are known as important marine food sources, from which phytosterols have been recognized as functional food components with multiple health-beneficial effects. However, studies on phytosterol extraction and quantitation from edible brown seaweeds are limited. In the present work, extraction methods for seaweed phytosterols were compared and optimized by one-factor-at-one-time method and response surface methodology. Moreover, the quantitation method of total sterols and major sterol components, including fucosterol, saringosterol, and ostreasterol, was established and validated using ^1^H NMR. Furthermore, the developed extraction and determination methods were applied to investigate three common edible seaweeds from Japan (Hijiki, Wakame, and Kombu). As a result, the finally optimized conditions were ultrasound-assisted extraction with CHCl_3_-MeOH 2:3 for 15 min followed by saponification with 1.65 mL of 1.85 M KOH for 14.5 h. Based on the developed methods, phytosterols in three seaweeds were compared, and Hijiki showed an abundant total sterol amount (2.601 ± 0.171 mg/g DW), significantly higher than Wakame (1.845 ± 0.137 mg/g DW) and Kombu (1.171 ± 0.243 mg/g DW). Notably, the composition of the sterol components varied in different seaweeds. These findings might help the nutritional investigation and functional food development concerning phytosterols from seaweeds.

## 1. Introduction

Edible brown seaweeds (Phaeophyceae) are important marine bioresources consumed as marine vegetables, health supplements, and even traditional medicines in East Asian countries, such as Japan, Korea, and China [1]. They have been reported to be rich in diverse bioactive secondary metabolites as health-beneficial constituents, typically dietary fibers, polysaccharides, amino acids, tocols, polyunsaturated fatty acids (PUFAs), and phytosterols [2,3,4]. Such a wide variety of products contribute to the development of the seaweed industry, which helps improve food security risks and earn economic interest [5]. Among these functional components, phytosterols have been of great interest for decades; they are well-known cholesterol-lowering phytochemicals with satisfactory safety [6,7,8,9]. Besides the hypolipidemic function, in recent years, the phytosterols isolated from brown seaweeds were found to possess other biological activities targeting chronic disorders, such as anticancer, anti-inflammatory, anti-photoaging, anti-osteoarthritic, immunomodulatory, hepatoprotection, antioxidation, and others [10,11,12,13]. Specifically, the representative brown seaweed-derived phytosterols are drawing a great interest. As the major constituents, fucosterol can minimize the inflammatory responses, suppress aging-induced endoplasmic reticulum stress, and protect the nervous system, and saringosterol shows a noticeable anti-atherosclerotic effect, while ostreasterol (24-methylene-cholesterol) is reported to prevent against cancers and inhibit neurodegenerative diseases [14,15,16,17,18]. Therefore, phytosterols in brown seaweeds will hopefully be a new source of functional foods and, thus, need to be systematically developed.

Most of the previous research on marine phytosterols focused on discovering the diversity of structures, evaluating bioactivities, and characterizing profiles (mostly semi-quantitative). Before functional food development and industrialization, it is always necessary to elucidate the chemical constitution (especially for those bioactive compounds from foodstuffs) and discover the health-beneficial functions via in vitro and in vivo studies. Not only the targeted molecules but also their analogs, derivatives, and metabolites are worth investigating [19]. However, as a fundamental procedure, the proper extraction of seaweed phytosterols is always neglected. In general, phytosterols are firstly extracted with organic solvents and then saponified to remove impurities (typically, fatty acids and chlorophylls) [10,11,20,21]. Until now, there has been no “standard” or entirely satisfactory protocol for phytosterol extraction, especially for those from brown seaweeds.

One of the key techniques in developing dietary functional nutrient components or health supplementation is the quantitation of phytosterols. At present, most phytosterol determination methods are based on chromatographic approaches, such as chromatography (GC) and liquid chromatography (LC) combined with chromogenic or spectrometric detection (with or without derivation) [22,23,24,25]. In our opinion, it is of great importance and necessity to know the total amount of all the phytosterols as well as the amount of the representative phytosterol constituents because, in addition to the three major phytosterols mentioned above, there are considerable minor phytosterols in edible brown seaweeds [14,26], of which the underlying functions are to be uncovered. Therefore, the ideal strategy should be a simultaneous measurement for multiple targets without chromatographic separation. As a rapid, robust, and non-invasive method, nuclear magnetic resonance (NMR) has been routinely used in the field of food sciences in recent years, which has been proven to be a practical tool to identify and determine chemical components in various samples [27,28,29]. It is also an advantage that one reference substance of known concentration (i.e., internal standard (IS)) can be used to analyze different targets in the complex matrix [30]. Recently, ^1^H NMR was successfully applied to two *Sargassum* seaweeds for measuring total phytosterols [31]. Nevertheless, a further investigation of the characteristic sterol constituents is desirable.

Herein, our current work aims to (1) establish a feasible quantitation method for phytosterol in brown seaweeds and (2) find an optimal procedure for achieving marine phytosterols. In this study, a comprehensive ^1^H NMR-based quantitation method for total sterols, fucosterol, saringosterol, and ostreasterol in three common edible brown seaweeds was developed, validated, and applied. Moreover, a series of conditions in extraction and purification were improved through a combined strategy using the one-factor-at-one-time (OFAT) and the design of experiments (DOE) methods. An ultrasound-assisted extraction coupled to a response surface methodology (RSM)-optimized purification process was discovered as the optimal approach to obtaining phytosterols. After that, the quantitation method development, validation, and application were carried out.

## 2. Materials and Methods

### 2.1. Chemicals

The authentic phytosterol standards, including fucosterol, saringosterol, and ostreasterol, were obtained from Sigma-Aldrich (Saint Louis, MO, USA), of which the purity was verified (estimated to be 96.1%, 98.3%, and 98.0% based on ^1^H NMR data, see Figure S1). For ^1^H NMR measurement, the internal standard (IS) 2,3,4,5-tetrachloronitrobenzene (>98.0%) was purchased from Tokyo Chemical Industry Co., Ltd. (Tokyo, Japan), while the solvent deuterated chloroform (CDCl_3_, >99.8%) was purchased from Kanto Chemical Co., Inc. (Tokyo, Japan). All the other chemicals were of the highest grade available and purchased from Sigma-Aldrich unless otherwise specified.

### 2.2. Seaweed Sample Collection

Three commonly consumed edible brown seaweeds, namely Hijiki (*Sargassum fusiforme*), Wakame (*Undaria pinnatifida*), and Kombu (*Saccharina japonica*), were investigated in the present work. All the seaweeds were collected in Japan from March to April 2021, of which the production area is listed in Table S1. The obtained edible part of the seaweed material was washed with distilled water, air-dried in the shade, and transported to the laboratory for the following processes. Next, the samples were ground into powder using a kitchen mill (BM-SS10-BA, Zojirushi Co., Ltd., Osaka, Japan) and filtered through a 20-mesh sieve. All the seaweed powder samples were stored at −80 °C under dry conditions until further treatments.

### 2.3. Optimization of Extraction Procedures

The basic routine was performed according to the previous literature [31], which generally initialized with total lipid extraction from raw seaweed samples, followed by saponification of total lipids to yield unsaponifiable matter (i.e., total phytosterols). Here in the extraction procedure, multiple parameters were optimized.

#### 2.3.1. Solvent System for Extraction

According to recent reports on phytosterol extraction, the following investigated solvent systems were selected for comparison: (1) chloroform/methanol [31,32], (2) hexane/2-propanol [33], (3) hexane/MTBE/2-propanol [34], and (4) ethanol [35]. For all the protocols, 750 mg of seaweed powder was used, and three extraction times were applied to ensure a sufficient yield [36].

1. Chloroform/methanol system (CHCl_3_-MeOH). The seaweed powder was added into 30 mL of chloroform/methanol (1:1, *v*/*v*), followed by vortex for 5 min. Next, 11 mL of water was added to the sample. The mixture was centrifuged, and the organic phase was dried under vacuum to yield the total lipids.
2. Hexane/2-propanol system (Hex-IPA). The seaweed powder was added into 30 mL of hexane/2-propanol (3:2, *v*/*v*), followed by vortex for 5 min. Next, the sample was centrifuged, and the supernatant was dried under vacuum to yield the total lipids.
3. Hexane/MTBE/2-propanol system (MTBE). The seaweed powder was added into 30 mL of hexane/MTBE/2-propanol (3:1:1, *v*/*v*/*v*), followed by vortex for 5 min. Next, the sample was centrifuged, and the supernatant was dried under vacuum to yield the total lipids.
4. Ethanol system (EtOH). The seaweed powder was added to 30 mL of ethanol, followed by vortex for 5 min. Next, the sample was centrifuged, and the supernatant was dried under vacuum to yield the total lipids.

Once the optimal system is chosen, the detailed solvent ratio will be investigated.

#### 2.3.2. Ultrasound-Assisted Extraction (UAE)

While conventional extraction techniques (e.g., vortex) have been widely used in seaweed studies, there are alternative and more advanced techniques. UAE has been known as an efficient, eco-friendly, and scalable technique for extracting bioactive components [37]. In this study, UAE was performed using a USC-6D ultrasonic processor (150 W, 38 kHz, UltrasonicEngineering Co., Ltd., Tokyo, Japan). The sample amount, number of extractions, solvent system, and volume were fixed to the same as vortex. For comparison, the extraction time was set as 1, 5, 10, and 15 min.

### 2.4. Optimization of Saponification Conditions Using RSM

Typically, saponification in phytosterol purification was performed in mild conditions with KOH-EtOH solution in the dark at room temperature [38,39]. In the present work, three variables were investigated, including the concentration of KOH, the time of reaction, and the volume of solution, which served as the critical factors of the saponification reaction. Therefore, a three-variable and five-level (−α, −1, 0, +1, +α) central composite design (CCD) experiment was employed to optimize the saponification conditions (Table S2), including KOH concentration (M, *X*_1_), reaction time (h, *X*_2_), and solution volume (mL, *X*_3_). All the variables were coded according to Equation (1). The complete experimental design with regard to each value in actual and the coded form is listed in Table 1, which contains 56 tests, 3 factorial points, 3 axial points, and 14 central points (1.5 M, 15 h, 1.5 mL), generated in random order. The amount and purity of the obtained seaweed phytosterols each were set as the response variable (*Y*), and the fitted polynomial equations were expressed as 3D response surface plots. The parameters R^2^, adjust-R^2^, variance analysis, and residuals analysis were employed to evaluate the model. The design and the response surface fit were conducted using the Design Expert 12.0 software (Stat-Ease, Inc., Minneapolis, MN, USA), and the optimal condition was obtained by further optimization of the expectation. After the reaction, the unsaponifiable matter was extracted with hexane and dried in vacuum to achieve total phytosterols.

### 2.5. H NMR Analysis and Processing

The dried unsaponifiable matter was dissolved with 500 µL of CDCl_3_ (containing 0.5 mg IS) for the NMR test. ^1^H NMR spectra were acquired on a JNM-ECX-400P spectrometer (400 MHz, JEOL Ltd., Tokyo, Japan). The optimal parameters were as below: pulse angle, 45°; relaxation delay (D1): 6.0 s; spectral width (SW), 5938.2 Hz; temperature, 298 K; scan times, 64. Free induction decays (FIDs) were processed with a line broadening (LB) of 0.2 Hz prior to Fourier transformation. Phase correction, baseline adjustment, and integration of the peak area were performed manually, and the chemical shifts of all the data were referenced to the IS resonance at *δ* 7.90. The peak area was used for the quantitative analysis, and the content of the phytosterols was calculated by the linear equation. All the NMR raw data processing was performed using MestReNova 12.0 (Mestrelab Research, S.L., Santiago de Compostela, Spain).

### 2.6. Validation of the ^1^H NMR Quantitation Method

The method validation was performed following the guidelines previously addressed by Japanese Pharmacopoeia Seventeenth Edition (JP17) [40]. Linearity was tested using a series of diluted PSs solutions. For sensitivity, limit of detection (LOD) and limit of quantification (LOQ) were separately determined at signal-to-noise ratio (S/N) values of 3 and 10, respectively. Repeatability, intermediate precision, and recovery were evaluated by six replicates (*n* = 6). Accuracy, reflected by recovery tests, was assessed at three levels (50%, 100%, and 200% of spiking, *n* = 6 for each).

### 2.7. Statistics

All the data are presented as the mean ± standard deviation (SD). Unpaired two-tailed *t*-test, one-way ANOVA, and two-way ANOVA (using the Tukey post hoc test) were conducted using GraphPad Prism 8.4 (GraphPad Software, Inc., La Jolla, CA, USA) and Excel 2021 (Microsoft Corporation, Redmond, WA, USA). *p* < 0.05 was considered statistically significant.

## 3. Results and Discussion

### 3.1. Detection of Phytosterols Using ^1^H NMR

**Figure 1:**
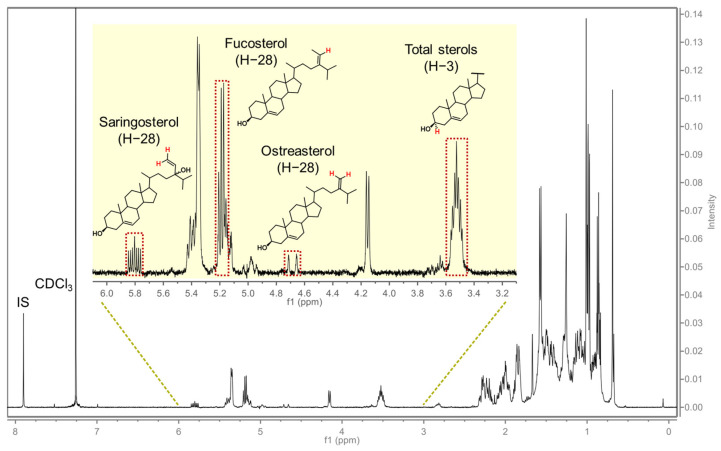
Typical ^1^H NMR spectrum of phytosterols in brown seaweed, taking Hijiki as an example.

The typical ^1^H NMR spectrum of brown seaweed phytosterols in our study is shown in Figure 1, in which *δ* 3.2–6.2 provided informative structural features of phytosterols, while *δ* 7.90 (*s*) conducted the resonance of IS. With the help of authentic standards, the characterized signals were assigned for the following targets: total phytosterol, *δ* 3.47–3.58 (*m*, H-3); fucosterol, *δ* 5.18 (*q*, *J* = 6.7 Hz, H-29); saringosterol, *δ* 5.80 (*ddd*, *J* = 16.6, 11.0, 5.8 Hz, H-28); and ostreasterol, *δ* 4.69 (*d*, *J* = 22.4 Hz, H-28). Such identical and specific NMR signals were fundamental to determining multiple sterols simultaneously. It is noted that our current results were consistent with a previous report [31] with regard to total sterols, while for the individual sterol analytes, it was the first time applying NMR to their quantitative analysis.

### 3.2. Optimization of Phytosterol Extraction Procedure by OFAT

#### 3.2.1. Comparing Different Extract Solvent Systems

**Figure 2:**
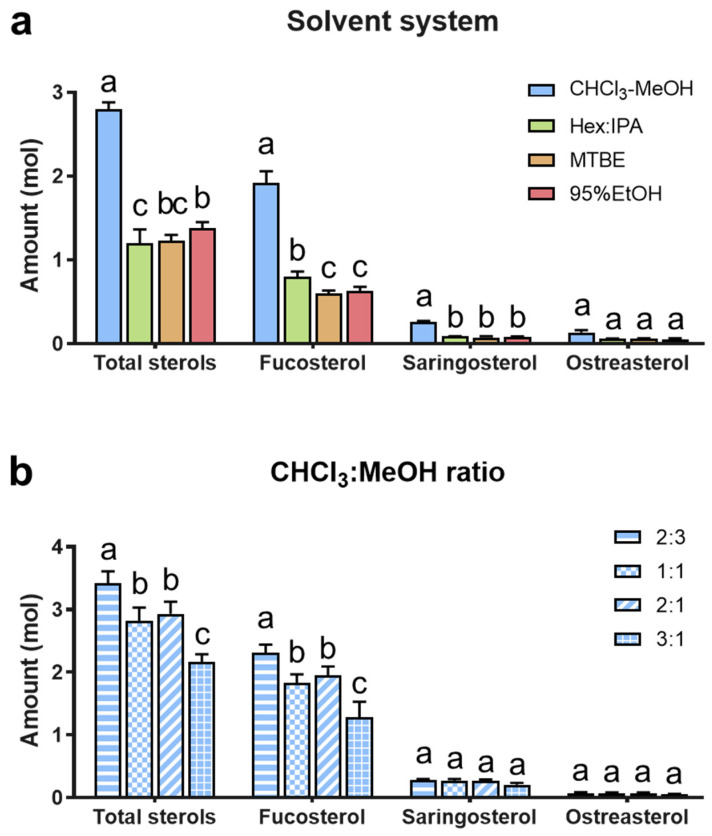
(**a**) Comparison of different extract solvent systems for extracting phytosterols, including chloroform/methanol (CHCl3-MeOH), hexane/2-propanol (Hex-IPA), hexane/MTBE/2-propanol (MTBE), and ethanol (EtOH). (**b**) Comparison of different chloroform-methanol ratios for extracting phytosterols, including 2:3, 1:1, 2:1, and 3:1. Bars that do not share similar letters denote statistical significance (*p* < 0.05). Data were analyzed by two-way ANOVA followed by Tukey’s multiple tests.

Initially, four commonly used extraction solvent systems were compared, which are shown in Figure 2a. The CHCl_3_-MeOH (1:1, *v*/*v*) solvent system resulted in the highest amount of phytosterols (*p* < 0.001 for all but ostreasterol), accounting for more than 2-fold that of other solvent systems. Although hexane–isopropanol, ethanol, and methyl tert-butyl ether systems were applied to extract seaweed phytosterols [32,33,39], the current results supported the classic CHCl_3_-MeOH system (with a one-phase extraction followed by a two-phase partition) as the most suitable solvent system, and thus, was worth further optimization.

Consequently, we tested different extracting solvents with the CHCl_3_-MeOH ratios of 2:3, 1:1, 2:1, and 3:1 (*v*/*v*) (Figure 2b). The results of total sterols and fucosterol suggested that CHCl_3_-MeOH 2:3 led to the highest yield (*p* < 0.05); moreover, there was a noticeable trend that the lower the solvent polarity, the less phytosterol extracted. However, according to our experiment, a higher proportion of MeOH caused the two-phase partition to be impossible. Therefore, the extreme value was set at 2:3. At the same time, there was no difference among these solvent ratios in saringosterol or ostreasterol, which might be due to their low amounts. Considering that fucosterol was the predominant sterol in brown seaweed [41], CHCl_3_-MeOH 2:3 was finally selected as the most suitable solvent to extract phytosterols from brown seaweeds.

#### 3.2.2. Confirming the Efficiency of UAE

**Figure 3:**
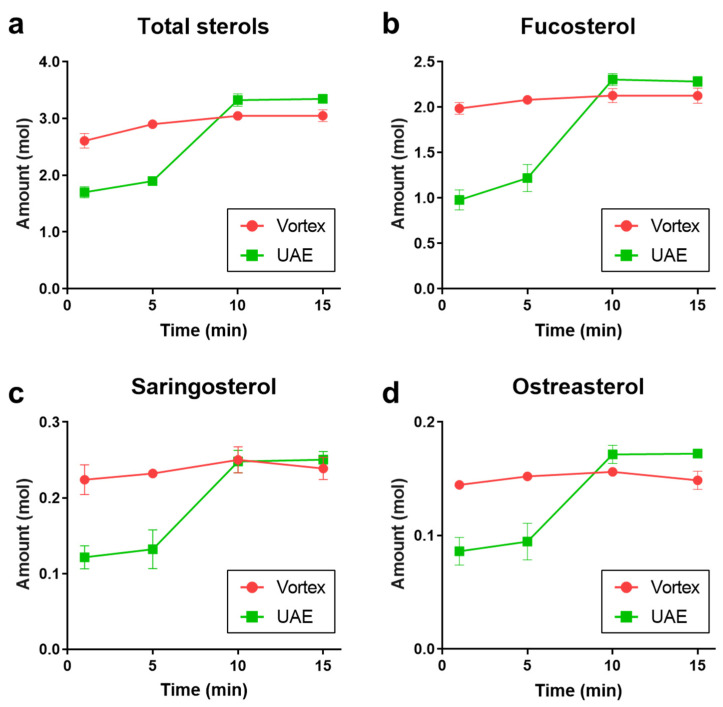
Comparison of extraction efficiency between vortex and UAE. (**a**) Total phytosterol; (**b**) Fucosterol; (**c**) Saringosterol; (**d**) Ostreasterol.

The efficiencies of the conventional vortex and the advanced extraction technique UAE were compared, as shown in Figure 3. Both methods showed the trend that a longer extraction time leads to increased phytosterol within 10 min. For a quick-time extraction, vortex was more effective than UAE: even 1 min could achieve 85.5–93.3% of the maximum yielded sterols. Meanwhile, for UAE, the watershed appeared between 5 min and 10 min: the later sterol yield accounted for 1.8–1.9 folds of the former. It is also noted that an extended extraction did not exhibit benefits for either total sterols or any specific phytosterols. Under the corresponding conditions, UAE obtained 9.7%, 7.3%, 4.9%, and 15.7% more than vortex for total sterols, fucosterol, saringosterol, and ostreasterol, respectively.

Therefore, the optimized extraction procedure was decided as UAE with CHCl_3_-MeOH 2:3 for 15 min.

### 3.3. Optimization of Saponification Procedure by RSM

The CCD was applied to optimize the saponification process for seaweed phytosterols and evaluate the effects of KOH concentration, reaction time, and solution volume. The resulted responses are listed in Table 2, including total sterol amount (*Y*_1_), fucosterol amount (*Y*_2_), saringosterol amount (*Y*_3_), and ostreasterol amount (*Y*_4_). Moreover, the results of ANOVA are shown in Tables S3–S6 as the evaluation of the predicted models, which revealed that the models were highly significant (*p* < 0.0001 for all), indicating that the models showed a good fit with our experimental data. Consequently, to explain the relationship between the response and the coded variables in the present work, the saponification yield values of phytosterols (*Y*_1_–*Y*_4_) could be described as the following four second-order polynomial Equations (1)–(4):

*Y*_1_ = 3.69658 + 0.25413*X*_1_ + 0.141448*X*_2_ + 0.725187*X*_3_ + 0.1545*X*_1_*X*_2_ − 0.21225*X*_1_*X*_3_ − 0.01875*X*_2_*X*_3_ − 0.220421*X*_1_^2^ − 0.169274*X*_2_^2^ − 0.617285*X*_3_^2^ (1)

*Y*_2_ = 2.42318 + 0.16124*X*_1_ + 0.119052*X*_2_ + 0.576618*X*_3_ + 0.0756667*X*_1_*X*_2_ − 0.258*X*_1_*X*_3_ + 0.0355*X*_2_*X*_3_ − 0.108244*X*_1_^2^ − 0.104355*X*_2_^2^ − 0.487607*X*_3_^2^ (2)

*Y*_3_ = 0.262579 + 0.00246853*X*_1_ − 0.00927938*X*_2_ + 0.0508653*X*_3_ + 0.00172917*X*_1_*X*_2_ − 0.0264792*X*_1_*X*_3_ − 0.0216875*X*_2_*X*_3_ − 0.000768389*X*_1_^2^ − 0.00129872*X*_2_^2^ − 0.0346506*X*_3_^2^ (3)

*Y*_4_ = 0.0987771 + 0.0126687*X*_1_ − 0.000619602*X*_2_ + 0.0217865*X*_3_ − 0.000416667*X*_1_*X*_2_ − 0.00225*X*_1_*X*_3_ − 0.00316667*X*_2_*X*_3_ − 0.00974362*X*_1_^2^ − 0.00989093*X*_2_^2^ − 0.0210279*X*_3_^2^ (4)

where *Y*_1_–*Y*_4_ represent the saponification yield of total sterols, fucosterol, saringosterol, and ostreasterol, respectively. For all the responses, the influence of the three single factors follows the order: solution volume > KOH concentration > reaction time.

**Figure 4:**
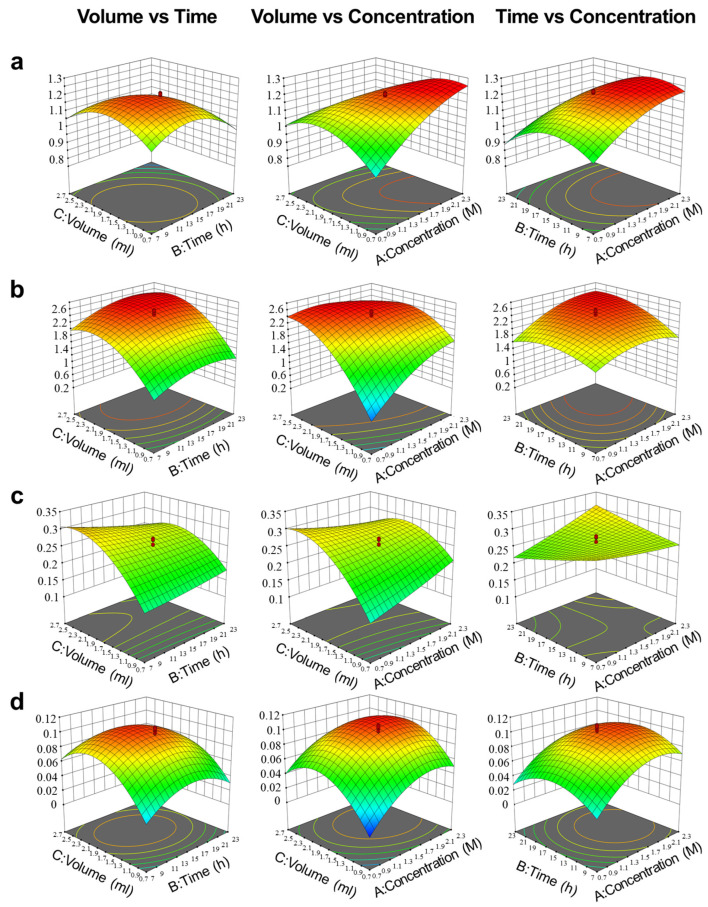
Response surface showing effects of independent variables (*X*_1_, concentration of KOH; *X*_2_, time of reaction; *X*_3_, volume of solution) on total phytosterol (**a**), fucosterol (**b**), saringosterol (**c**), and ostreasterol (**d**).

The effects of independent variables on yield were illustrated by three-dimensional response surface plots (Figure 4). KOH concentration was the most significant variable for total sterols: the yield elevated along with the concentration increased from 0.7 to 2.3 M, while for saringosterol, a more concentrated KOH did not result in any improvement. As another critical factor of saponification, reaction time was suitable to be within 12–16 h for total sterols, fucosterol, and ostreasterol, but it seemed not to affect saringosterol. It was reported that fucosterol could be formed into saringosterol via spontaneous oxidative degradation [42]. In our study, the results of saringosterol were not similar to fucosterol or ostreasterol, which might suggest that saringosterol yield was not only related to purification through saponification but also involved in production as an artifact.

Since not only total sterols but also each phytosterol constituent was desired, the final optimal saponification condition was obtained by numerical expectation function method and decided as follows: KOH concentration, 1.85 M; reaction time, 14.5 h; solution volume, 1.65 mL. Subsequently, for testing the prediction accuracy of the model, six replicates of the optimum points were prepared and analyzed, which were in agreement with the predicted value, indicating that the optimized model was capable of predicting the actual purification process of seaweed phytosterols.

**Figure 5:**
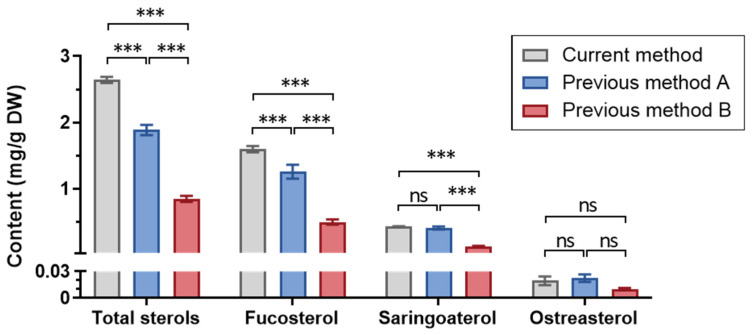
Comparison of currently optimized and previous methods for the obtained content of total sterols, fucosterol, saringosterol, and ostreasterol. *** *p* < 0.001, analyzed by two-way ANOVA followed by Tukey’s multiple tests.

The currently optimized method was compared with the typical methods reported previously (A, a modified Folch’s method [31]; B, a 95% EtOH-based method [43]). Taking the Hijiki No.4 sample as an example, the current method resulted in 2.642 ± 0.046 mg/g DW of total sterols, which equaled 1.4-fold of method A (1.887 ± 0.077 mg/g DW), or 3.1-fold of method B (0.849 ± 0.047 mg/g DW). For fucosterol, similar findings were observed: compared with methods A (1.258 ± 0.106 mg/g DW) and B (0.499 ± 0.040 mg/g DW), the current method (1.598 ± 0.047 mg/g DW) increased the yield by 27.0% and 220.2%, respectively. While for saringosterol and ostreasterol, the current method and method A showed comparable effects, which were slightly higher than method B (Figure 5). Although method B required ethanol as the organic solvent, which was green and suitable for industrial applications, chloroform-based methods exhibited much higher efficiency for phytosterol accumulation, which benefitted the studies on the material basis of seaweed phytosterols. These data confirmed that seaweed phytosterols could be more effectively achieved through the comprehensive optimization of extraction and purification.

### 3.4. Validation of the Phytosterol Quantitation Method

Method validation was conducted according to JP17 [40], as shown in Table 3. The calibration curves for all these analytes (total sterols, fucosterol, saringosterol, and ostreasterol) showed satisfied R^2^ (>0.998 for all) within their linear ranges. The sensitivity of this method was sufficient for this work: the LOQ of the four analytes ranged from 7.50 µg (ostreasterol) to 93.8 µg (fucosterol), and the LOD ranged from 15.00 µg (ostreasterol) to 187.5 µg (fucosterol). For precision, the CV of each analyte obtained was below 6% of both intra-assay precision and intermediate precision, which fit the requirements of JP17. In terms of accuracy, the recovery of each analyte in all three levels was within the range of 86.1–101.7%. These data proved that the developed determination method using ^1^H NMR was validated and practical for quantitatively investigating phytosterols in edible brown seaweeds.

Although there have recently been reports of phytosterol determination applied to food sciences using chromatographic approaches (i.e., LC/MS or GC/MS) [25,44,45], our method based on ^1^H NMR expressed satisfied linearity and sensitivity, as well as sufficient precision and accuracy, which was comparable with MS-based methods. Moreover, the current method avoided separation and detected the total phytosterols from a holistic perspective. Therefore, 1H NMR-based quantitation was considered more suitable for this work than chromatographic approaches.

### 3.5. Application of the Extraction and Quantitation Methods to Seaweed Samples

**Figure 6:**
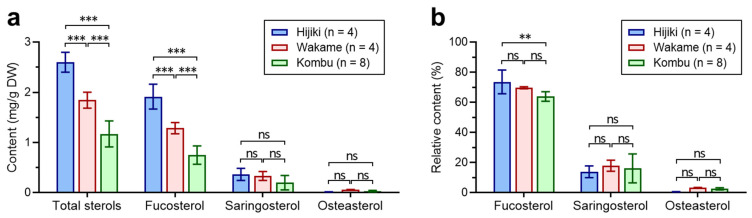
Comparison of the phytosterols in three common edible seaweeds: (**a**) Content of total sterols, fucosterol, saringosterol, and ostreasterol. (**b**) Relative content of phytosterol components. ** *p* < 0.01, *** *p* < 0.001, analyzed by one-way ANOVA followed by Tukey’s multiple tests.

We applied the established methods to 16 batches of three commonly consumed brown seaweeds, including 4 batches of Hijiki, 4 batches of Wakame, and 8 batches of Kombu. The data are shown in Table S7, and the comparison of sterol content and composition is presented in Figure 6. The total phytosterol content varied among different kinds of seaweeds significantly (*p* < 0.05): Hijiki exhibited the highest total sterol amount (2.601 ± 0.171 mg/g DW), followed by Wakame (1.845 ± 0.137 mg/g DW), while Kombu showed the lowest (1.171 ± 0.243 mg/g DW). For each phytosterol constituent, the content of fucosterol expressed the same trend as total sterols, i.e., Hijiki > Wakame > Kombu (*p* < 0.05 for all); while saringosterol did not show significant distinction in the investigated samples.

In terms of phytosterol composition, fucosterol served as the predominant sterol (73.6% ± 6.8% in Hijiki, 69.7% ± 0.6% in Wakame, and 64.0% ± 3.0% in Kombu), which was consistent with previous studies [10]. Although Hijiki contained the highest percentage of fucosterol, it showed the lowest saringosterol (13.9% ± 3.3%) and ostreasterol (0.7% ± 0.1%) compared with the other two seaweeds (saringosterol: 17.9% ± 3.2% in Wakame and 16.2% ± 9.0% in Kombu; ostreasterol: 3.3% ± 0.2 in Wakame and 2.6% ± 0.7% in Kombu). It was also noted that even within the same seaweed, the content and composition of phytosterol broadly varied, which might be due to cultivation or environmental factors, such as temperature, sunlight, and ocean currents [46,47]. Therefore, further systematic investigations on the variations of phytosterol profiles in different seaweed samples are needed in the future.

Importantly, there was an apparent shortage of the current method: it was a chloroform-based method, which was not environment-friendly and unsuitable for the food industry. In the present study, we were focused on the material basis of edible brown seaweeds, and we proposed to perform phytosterol resource screening, bioactivity evaluations (in vitro or in vivo experiments), semi-synthesis and other chemical modifications, and bioavailability studies. Therefore, an optimal way to accumulate natural marine phytosterols became fundamental. Concerning industrial production, a green, safe, and economical extraction procedure needs to be developed in the future. Nevertheless, the present findings could provide essential information on edible seaweeds with respect to nutritional evaluation, the marine food industry, and health supplement development. Moreover, the established extraction and quantitation methods can hopefully be applied to comprehensive studies on phytosterol functional foods.

## 4. Conclusions

In summary, the current study compared and optimized the extraction methods for phytosterols from edible brown seaweeds by OFAT and RSM-based DOE; then, developed and validated the phytosterol quantitation method using ^1^H NMR; and finally, applied this method to commonly consumed seaweeds. For the current study, the optimal procedure was found to be UAE with CHCl_3_-MeOH 2:3 for 15 min as the extraction, followed by saponification with 1.65 mL of 1.85 M KOH for 14.5 h. The developed method was proven reliable and feasible for seaweed phytosterol quantitation. Moreover, a comparison of phytosterols in Hijiki, Wakame, and Kombu revealed specific sterol content and composition. Additionally, this method could be applied to accumulate phytosterols from foodstuffs for biological assays and chemical modifications. Therefore, our present work might contribute to further studies on developing extended phytosterol functional food sources.

## Figures and Tables

**Table 1:** Coded and natural values of central composite design (CCD) for the independent variables.

Run	Conc. (M) (*X*_1_)		T. (h) (*X*_2_)		Vol. (mL) (*X*_3_)		Run	Conc. (M) (*X*_1_)		T. (h) (*X*_2_)		Vol. (mL) (*X*_3_)	
1	+1	(2)	−1	(10)	−1	(0.75)	29	−1	(1)	+1	(20)	−1	(0.75)
2	+1	(2)	+1	(20)	−1	(0.75)	30	−α	(0.659)	0	(15)	0	(1.5)
3	0	(1.5)	0	(15)	−α	(0.239)	31	0	(1.5)	0	(15)	0	(1.5)
4	−1	(1)	−1	(10)	+1	(2.25)	32	0	(1.5)	0	(15)	0	(1.5)
5	+1	(2)	+1	(20)	−1	(0.75)	33	−α	(0.659)	0	(15)	0	(1.5)
6	+1	(2)	+1	(20)	+1	(2.25)	34	0	(1.5)	0	(15)	0	(1.5)
7	0	(1.5)	0	(15)	0	(1.5)	35	+α	(2.34)	0	(15)	0	(1.5)
8	0	(1.5)	0	(15)	0	(1.5)	36	−1	(1)	+1	(20)	−1	(0.75)
9	−1	(1)	−1	(10)	+1	(2.25)	37	0	(1.5)	0	(15)	0	(1.5)
10	+1	(2)	+1	(20)	−1	(0.75)	38	−α	(0.659)	0	(15)	0	(1.5)
11	0	(1.5)	+α	(23.4)	0	(1.5)	39	−1	(1)	−1	(10)	−1	(0.75)
12	−1	(1)	−1	(10)	−1	(0.75)	40	+1	(2)	1	(20)	+1	(2.25)
13	+1	(2)	−1	(10)	+1	(2.25)	41	0	(1.5)	0	(15)	−α	(0.239)
14	0	(1.5)	0	(15)	+α	(2.76)	42	−1	(1)	+1	(20)	+1	(2.25)
15	0	(1.5)	0	(15)	0	(1.5)	43	0	(1.5)	0	(15)	0	(1.5)
16	−1	(1)	+1	(20)	+1	(2.25)	44	+1	(2)	+1	(20)	+1	(2.25)
17	0	(1.5)	0	(15)	+α	(2.76)	45	−1	(1)	+1	(20)	+1	(2.25)
18	+α	(2.34)	0	(15)	0	(1.5)	46	0	(1.5)	0	(15)	0	(1.5)
19	0	(1.5)	0	(15)	+α	(2.76)	47	0	(1.5)	0	(15)	0	(1.5)
20	0	(1.5)	+α	(23.4)	0	(1.5)	48	0	(1.5)	−α	(6.59)	0	(1.5)
21	0	(1.5)	0	(15)	−α	(0.239)	49	0	(1.5)	0	(15)	0	(1.5)
22	+α	(2.34)	0	(15)	0	(1.5)	50	0	(1.5)	−α	(6.59)	0	(1.5)
23	0	(1.5)	0	(15)	0	(1.5)	51	0	(1.5)	+α	(23.4)	0	(1.5)
24	+1	(2)	−1	(10)	+1	(2.25)	52	0	(1.5)	−α	(6.59)	0	(1.5)
25	+1	(2)	−1	(10)	+1	(2.25)	53	+1	(2)	−1	(10)	−1	(0.75)
26	−1	(1)	−1	(10)	+1	(2.25)	54	0	(1.5)	0	(15)	0	(1.5)
27	−1	(1)	−1	(10)	−1	(0.75)	55	+1	(2)	−1	(10)	−1	(0.75)
28	−1	(1)	+1	(20)	−1	(0.75)	56	0	(1.5)	0	(15)	0	(1.5)

Conc., concentration of KOH; T., time of reaction; Vol., volume of solution.

**Table 2:** Results of CCD for the saponification procedure.

9	Variables			Responses				Run	Variables			Responses			
	*X*_1_	*X*_2_	*X*_3_	*Y*_1_	*Y*_2_	*Y*_3_	*Y*_4_		*X*_1_	*X*_2_	*X*_3_	*Y*_1_	*Y*_2_	*Y*_3_	*Y*_4_
1	1.5	15	1.5	3.77	2.40	0.277	0.105	29	1.5	6.59	1.5	3.21	2.23	0.256	0.084
2	1	10	0.75	1.64	0.43	0.199	0.011	30	0.659	15	1.5	2.85	2.29	0.249	0.042
3	2	20	2.25	3.81	2.35	0.200	0.090	31	1.5	15	0.239	0.58	0.05	0.030	0.002
4	1.5	23.4	1.5	3.42	2.27	0.210	0.074	32	2	10	2.25	3.07	1.68	0.380	0.091
5	1.5	15	1.5	3.73	2.48	0.260	0.092	33	1.5	15	1.5	3.78	2.17	0.277	0.091
6	1.5	15	1.5	3.75	2.55	0.258	0.110	34	1.5	15	1.5	3.68	2.57	0.254	0.098
7	1	10	2.25	3.12	2.22	0.382	0.043	35	0.659	15	1.5	2.99	2.19	0.255	0.091
8	1.5	23.4	1.5	3.23	2.26	0.246	0.075	36	1.5	15	1.5	3.73	2.43	0.261	0.095
9	2	10	0.75	2.28	1.31	0.227	0.042	37	1	20	0.75	1.60	0.59	0.148	0.011
10	2.34	15	1.5	3.28	2.29	0.244	0.097	38	1	10	2.25	3.13	1.84	0.251	0.061
11	1.5	6.59	1.5	3.21	2.17	0.251	0.086	39	1.5	15	1.5	3.61	2.38	0.263	0.083
12	1.5	15	1.5	3.71	2.13	0.262	0.108	40	1	10	2.25	3.22	1.94	0.306	0.059
13	1.5	15	1.5	3.47	2.41	0.231	0.082	41	1.5	15	2.76	3.38	2.31	0.273	0.093
14	1	20	2.25	3.15	2.34	0.269	0.069	42	1	20	2.25	3.19	2.29	0.275	0.055
15	2	20	2.25	3.84	2.51	0.200	0.066	43	1.5	15	2.76	3.43	2.29	0.290	0.088
16	2	10	2.25	3.00	2.20	0.256	0.093	44	1.5	23.4	1.5	3.36	2.20	0.270	0.067
17	1.5	15	1.5	3.72	2.39	0.278	0.102	45	2	10	2.25	3.22	1.77	0.253	0.091
18	2.34	15	1.5	3.22	2.27	0.257	0.082	46	1	10	0.75	1.08	0.51	0.153	0.012
19	1.5	15	2.76	3.32	2.14	0.271	0.094	47	1.5	15	0.239	0.70	0.03	0.027	0.001
20	2	10	0.75	2.27	1.38	0.195	0.058	48	1.5	6.59	1.5	3.15	2.22	0.242	0.083
21	1.5	15	1.5	3.57	2.55	0.264	0.108	49	2.34	15	1.5	3.30	2.17	0.229	0.080
22	2	20	0.75	2.97	2.02	0.266	0.074	50	2	20	2.25	3.92	2.56	0.200	0.093
23	1	20	0.75	1.58	0.77	0.178	0.027	51	1.5	15	1.5	3.79	2.50	0.281	0.105
24	1.5	15	0.239	0.56	0.03	0.018	0.001	52	2	10	0.75	2.16	1.53	0.210	0.048
25	1	20	0.75	1.39	0.70	0.156	0.014	53	0.659	15	1.5	3.07	2.07	0.250	0.079
26	2	20	0.75	2.94	1.85	0.259	0.070	54	1	10	0.75	1.21	0.68	0.180	0.012
27	2	20	0.75	2.89	1.84	0.247	0.042	55	1	20	2.25	3.14	2.39	0.254	0.045
28	1.5	15	1.5	3.43	2.30	0.471	0.093	56	1.5	15	1.5	3.47	2.19	0.231	0.080

*X*_1_, concentration of KOH (M); *X*_2_, time of reaction (h); *X*_3_, volume of solution (mL); *Y*_1_, total sterol amount (mol); *Y*_2_, fucosterol amount (mol); *Y*_3_, saringosterol amount (mol); *Y*_4_, ostreasterol amount (mol).

**Table 3:** Linearity, sensitivity, precision, and accuracy of the phytosterol analytes.

Validation Characteristics		Total Sterols	Fucosterol	Saringosterol	Ostreasterol
Linearity	Equation	y = 0.5761x − 0.3626	y = 1.0201x − 0.0308	y = 0.5997x − 0.0013	y = 0.1163x − 0.0013
	Linear range (µg)	210.9–6750	187.5–6000	23.4–750	7.5–240
	R^2^	0.9989	0.9999	0.9999	0.9999
Sensitivity	LOD (µg)	93.75	187.50	93.75	15.00
	LOQ (µg)	46.88	93.80	46.88	7.50
Precision	Intra-assay precision CV	2.0%	1.8%	2.6%	5.6%
	Intermediate precision CV	0.5%	3.8%	4.6%	5.8%
Accuracy	Recovery (50% spiking level)	97.3% ± 1.8%	95.0% ± 1.6%	86.1% ± 3.1%	89.8% ± 0.9%
	Recovery (100% spiking level)	99.0% ± 4.6%	99.2% ± 1.4%	91.0% ± 6.3%	91.1% ± 3.2%
	Recovery (200% spiking level)	101.3% ± 0.5%	101.7% ± 3.3%	90.9% ± 1.8%	93.7% ± 2.0%

## Data Availability

Data are contained within the article or Supplementary Materials.

## Supplementary Materials

The following supporting information can be downloaded at: https://www.mdpi.com/article/10.3390/foods12020244/s1, Figure S1. ^1^H NMR spectra of fucosterol (a), saringosterol (b), and ostreasterol (c) standards; Table S1: Information of the investigated brown seaweed samples; Table S2: Central composite design with three-variables and five-levels; Table S3: ANOVA for total sterols as the response; Table S4: ANOVA for fucosterol as the response; Table S5: ANOVA for saringosterol as the response; Table S6: ANOVA for ostreasterol as the response; Table S7: Phytosterol content in edible brown seaweed samples investigated (means ± SD).
