# Supplementary material for: Extraction and Quantitation of Phytosterols from Edible Brown Seaweeds: Optimization, Validation, and Application
Source: Foods. 2023 Jan 5;12(2):244. doi: 10.3390/foods12020244 (PMC9858231; doi:10.3390/foods12020244)
Supplement: Supplementary file 1 [file foods-12-00244-s001.zip › foods-2079412-supplementary.docx]

Extraction and Quantitation of Phytosterols from Edible Brown Seaweeds: Optimization, Validation, and Application

Zhen Chen ^1,†^, Nianqiu Shen ^1,†^, Xunzhi Wu ^1^, Jiaping Jia ^1^, Yue Wu ^1^, Hitoshi Chiba ^2^ and Shu-Ping Hui ^1,^*

^1^ Faculty of Health Sciences, Hokkaido University, Kita-12, Nishi-5, Kita-Ku, Sapporo 060-0812, Japan

^2^ Department of Nutrition, Sapporo University of Health Sciences, Nakanuma Nishi-4-2-1-15, Higashi-Ku, Sapporo 007-0894, Japan

***** Correspondence: keino@hs.hokudai.ac.jp; Tel./Fax: +81-11-706-3693

^†^ These authors contributed equally to this work.


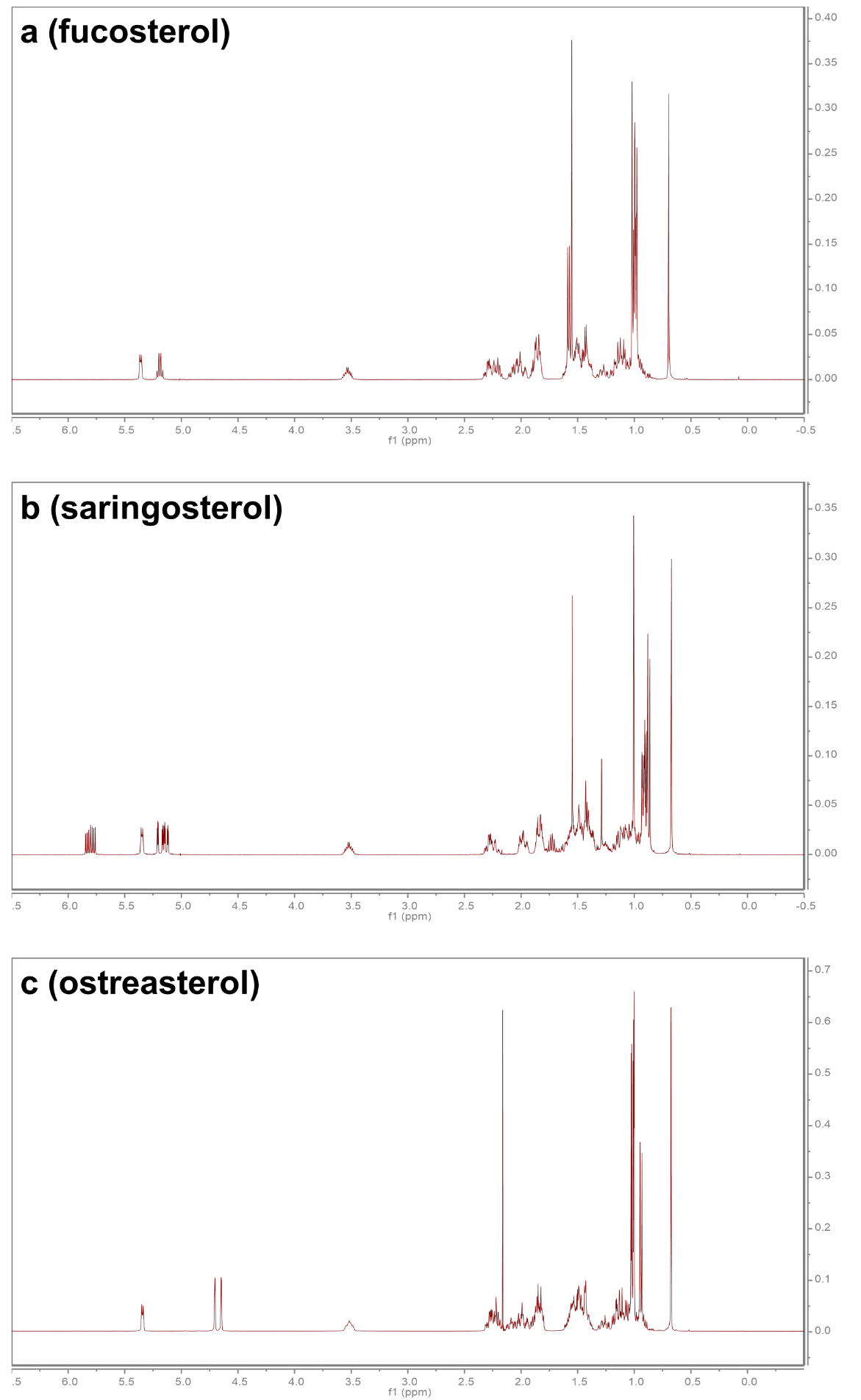


Figure S1. ^1^H NMR spectra of fucosterol (**a**), saringosterol (**b**), and ostreasterol (**c**) standards.

Table S1. Information of the investigated brown seaweed samples

| Brown Seaweeds | Sample numbers (n) | Production Area | | Code |
| --- | --- | --- | --- | --- |
| Hijiki  (*Sargassum fusiforme*) | 4 | Minamibousou, Chiba | 35°01′N 139°50′E | 1 |
|  |  | Kesennuma, Miyagi | 38°51′N 141°35′E | 2 |
|  |  | Ise, Mie | 34°31′N 136°44′E | 3 |
|  |  | Kikonai, Hokkaido | 41°42′N 140°32′E | 4 |
| Wakame  (*Undaria pinnatifida*) | 4 | Miyako, Iwate | 39°45′N 141°59′E | 5 |
|  |  | Ishinomaki, Miyagi | 38°23′N 141°30′E | 6 |
|  |  | Hakodate, Hokkaido | 41°53′N 141°03′E | 7 |
|  |  | Minamisanriku, Miyagi | 38°42′N 141°31′E | 8 |
| Kombu  (*Saccharina japonica*) | 8 | Hakodate, Hokkaido | 41°44′N 141°04′E | 9 |
|  |  | Kushiro, Hokkaido | 42°56′N 144°26′E | 10 |
|  |  | Hidaka, Hokkaido | 42°26′N 142°12′E | 11 |
|  |  | Rausu, Hokkaido | 44°11′N 145°19′E | 12 |
|  |  | Hakodate, Hokkaido | 41°46′N 140°46′E | 13 |
|  |  | Hidaka, Hokkaido | 42°36′N 141°49′E | 14 |
|  |  | Matsumae, Hokkaido | 41°36′N 139°59′E | 15 |
|  |  | Rishiri, Hokkaido | 45°06′N 141°17′E | 16 |

Table S2. Central composite design with three-variables and five-levels

| Variable | Units | Low | High | −α | +α |
| --- | --- | --- | --- | --- | --- |
| Concentration of KOH | M | 1 | 2 | 0.659 | 2.34 |
| Time of reaction | h | 10 | 20 | 6.591 | 23.4 |
| Volume of solution | mL | 0.75 | 2.25 | 0.239 | 2.76 |

Table S3. ANOVA for total sterols as the response^a,b^

| Source | Sum of Squares | Df | Mean Square | F-value | p-value |
| --- | --- | --- | --- | --- | --- |
| Model | 41.47 | 9 | 4.61 | 110.26 | < 0.0001 |
| A-Concentration | 2.62 | 1 | 2.62 | 62.80 | < 0.0001 |
| B-Time | 0.8197 | 1 | 0.8197 | 19.61 | < 0.0001 |
| C-Volume | 21.55 | 1 | 21.55 | 515.54 | < 0.0001 |
| AB | 0.5729 | 1 | 0.5729 | 13.71 | 0.0006 |
| AC | 1.08 | 1 | 1.08 | 25.87 | < 0.0001 |
| BC | 0.0084 | 1 | 0.0084 | 0.2019 | 0.6554 |
| A² | 1.87 | 1 | 1.87 | 44.69 | < 0.0001 |
| B² | 1.09 | 1 | 1.09 | 26.01 | < 0.0001 |
| C² | 14.46 | 1 | 14.46 | 345.94 | < 0.0001 |
| Residual | 1.84 | 44 | 0.0418 |  |  |
| Lack of Fit | 1.43 | 5 | 0.2860 | 27.28 | < 0.0001 |
| Pure Error | 0.4088 | 39 | 0.0105 |  |  |
| Cor Total | 43.31 | 53 |  |  |  |

^a^ R^2^: 0.9575, Adj. R^2^: 0.9489, Pred. R^2^: 0.9324, Std. Dev. = 0.204, CV = 6.97%, PRESS = 2.93.

^b^ *P* < 0.05 indicates that the model terms are significant.

Table S4. ANOVA for fucosterol as the response^a,b^

| Source | Sum of Squares | Df | Mean Square | F-value | p-value |
| --- | --- | --- | --- | --- | --- |
| Model | 26.06 | 9 | 2.90 | 62.64 | < 0.0001 |
| A-Concentration | 1.07 | 1 | 1.07 | 23.05 | < 0.0001 |
| B-Time | 0.5807 | 1 | 0.5807 | 12.56 | 0.0009 |
| C-Volume | 13.62 | 1 | 13.62 | 294.74 | < 0.0001 |
| AB | 0.1374 | 1 | 0.1374 | 2.97 | 0.0917 |
| AC | 1.60 | 1 | 1.60 | 34.56 | < 0.0001 |
| BC | 0.0302 | 1 | 0.0302 | 0.6544 | 0.4229 |
| A² | 0.4446 | 1 | 0.4446 | 9.62 | 0.0034 |
| B² | 0.4132 | 1 | 0.4132 | 8.94 | 0.0046 |
| C² | 9.02 | 1 | 9.02 | 195.21 | < 0.0001 |
| Residual | 2.03 | 44 | 0.0462 |  |  |
| Lack of Fit | 1.41 | 5 | 0.2826 | 17.77 | < 0.0001 |
| Pure Error | 0.6204 | 39 | 0.0159 |  |  |
| Cor Total | 28.09 | 53 |  |  |  |

^a^ R^2^: 0.9276, Adj. R^2^: 0.9128, Pred. R^2^: 0.8863, Std. Dev. = 0.215, CV = 11.36%, PRESS = 3.19.

^b^ *P* < 0.05 indicates that the model terms are significant.

Table S5. ANOVA for saringosterol as the response^a,b^

| Source | Sum of Squares | Df | Mean Square | F-value | p-value |
| --- | --- | --- | --- | --- | --- |
| Model | 0.1862 | 9 | 0.0207 | 16.42 | < 0.0001 |
| A-Concentration | 0.0002 | 1 | 0.0002 | 0.1982 | 0.6584 |
| B-Time | 0.0035 | 1 | 0.0035 | 2.80 | 0.1013 |
| C-Volume | 0.1060 | 1 | 0.1060 | 84.15 | < 0.0001 |
| AB | 0.0001 | 1 | 0.0001 | 0.0570 | 0.8125 |
| AC | 0.0168 | 1 | 0.0168 | 13.36 | 0.0007 |
| BC | 0.0113 | 1 | 0.0113 | 8.96 | 0.0045 |
| A² | 0.0000 | 1 | 0.0000 | 0.0178 | 0.8945 |
| B² | 0.0001 | 1 | 0.0001 | 0.0508 | 0.8227 |
| C² | 0.0456 | 1 | 0.0456 | 36.17 | < 0.0001 |
| Residual | 0.0554 | 44 | 0.0013 |  |  |
| Lack of Fit | 0.0291 | 5 | 0.0058 | 8.63 | < 0.0001 |
| Pure Error | 0.0263 | 39 | 0.0007 |  |  |
| Cor Total | 0.2416 | 53 |  |  |  |

^a^ R^2^: 0.7706, Adj. R^2^: 0.7236, Pred. R^2^: 0.6298, Std. Dev. = 0.0355, CV = 15.12%, PRESS = 0.0894.

^b^ *P* < 0.05 indicates that the model terms are significant.

Table S6. ANOVA for ostreasterol as the response^a,b^

| Source | Sum of Squares | Df | Mean Square | F-value | p-value |
| --- | --- | --- | --- | --- | --- |
| Model | 0.0452 | 9 | 0.0050 | 24.70 | < 0.0001 |
| A-Concentration | 0.0066 | 1 | 0.0066 | 32.36 | < 0.0001 |
| B-Time | 0.0000 | 1 | 0.0000 | 0.0774 | 0.7821 |
| C-Volume | 0.0194 | 1 | 0.0194 | 95.72 | < 0.0001 |
| AB | 4.167E-06 | 1 | 4.167E-06 | 0.0205 | 0.8868 |
| AC | 0.0001 | 1 | 0.0001 | 0.5980 | 0.4435 |
| BC | 0.0002 | 1 | 0.0002 | 1.18 | 0.2824 |
| A² | 0.0036 | 1 | 0.0036 | 17.73 | 0.0001 |
| B² | 0.0037 | 1 | 0.0037 | 18.27 | 0.0001 |
| C² | 0.0168 | 1 | 0.0168 | 82.59 | < 0.0001 |
| Residual | 0.0089 | 44 | 0.0002 |  |  |
| Lack of Fit | 0.0045 | 5 | 0.0009 | 7.99 | < 0.0001 |
| Pure Error | 0.0044 | 39 | 0.0001 |  |  |
| Cor Total | 0.0541 | 53 |  |  |  |

^a^ R^2^: 0.8348, Adj. R2: 0.8010, Pred. R2: 0.7417, Std. Dev. = 0.0143, CV = 20.98%, PRESS = 0.014.

^b^ *P* < 0.05 indicates that the model terms are significant.

Table S7. Phytosterol content in edible brown seaweed samples investigated (means ± SD).

| Seaweed | Sample  code | Total sterols | Fucosterol | | Saringosterol | | Ostreasterol | |
| --- | --- | --- | --- | --- | --- | --- | --- | --- |
|  |  | Content  (mg/g) | Content  (mg/g) | Percentage | Content  (mg/g) | Percentage | Content  (mg/g) | Percentage |
| Hijiki | 1 | 2.430 | 1.902 | 78.25% | 0.217 | 8.95% | 0.019 | 0.79% |
|  | 2 | 2.510 | 1.960 | 78.10% | 0.322 | 12.83% | 0.016 | 0.62% |
|  | 3 | 2.883 | 2.199 | 76.27% | 0.480 | 16.64% | 0.017 | 0.58% |
|  | 4 | 2.580 | 1.597 | 61.88% | 0.447 | 17.31% | 0.018 | 0.68% |
| *Average* | | 2.601 ± 0.198 | 1.914 ± 0.248 | 73.62% ± 7.88% | 0.366 ± 0.120 | 13.93% ± 3.86% | 0.017 ± 0.002 | 0.67% ± 0.09% |
| Wakame | 5 | 1.959 | 1.351 | 68.97% | 0.428 | 21.86% | 0.059 | 3.01% |
|  | 6 | 1.623 | 1.133 | 69.83% | 0.217 | 13.37% | 0.057 | 3.48% |
|  | 7 | 1.960 | 1.383 | 70.58% | 0.330 | 16.83% | 0.067 | 3.40% |
|  | 8 | 1.837 | 1.277 | 69.48% | 0.361 | 19.66% | 0.059 | 3.21% |
| *Average* | | 1.845 ± 0.159 | 1.286 ± 0.111 | 69.72% ± 0.68% | 0.334 ± 0.088 | 17.93% ± 3.67% | 0.060 ± 0.004 | 3.27% ± 0.21% |
| Kombu | 9 | 1.180 | 0.790 | 66.98% | 0.154 | 13.05% | 0.033 | 2.84% |
|  | 10 | 1.494 | 0.900 | 60.23% | 0.374 | 25.03% | 0.055 | 3.65% |
|  | 11 | 1.197 | 0.746 | 62.33% | 0.105 | 8.80% | 0.032 | 2.66% |
|  | 12 | 0.926 | 0.554 | 59.78% | 0.269 | 29.01% | 0.022 | 2.40% |
|  | 13 | 1.584 | 1.093 | 69.03% | 0.441 | 27.83% | 0.051 | 3.24% |
|  | 14 | 1.167 | 0.752 | 64.44% | 0.138 | 11.82% | 0.031 | 2.63% |
|  | 15 | 0.906 | 0.590 | 65.14% | 0.041 | 4.56% | 0.012 | 1.32% |
|  | 16 | 0.916 | 0.584 | 63.82% | 0.085 | 9.33% | 0.020 | 2.24% |
| *Average* | | 1.171 ± 0.259 | 0.751 ± 0.183 | 63.97% ± 3.17% | 0.201 ± 0.145 | 16.18% ± 9.59% | 0.032 ± 0.015 | 2.62% ± 0.70% |
